# Observations upon Some of the Medical Plants Mentioned by Shakspeare

**Published:** 1833-08

**Authors:** Samuel Rootsey

**Affiliations:** Corresponding Member of the Medico-Botanical Society.


### MEDICAL PLANTS MENTIONED BY SHAKSPEARE.

*Observations upon some of the Medical Plants mentioned by Shakspeare.* Communicated by Samuel Rootsey, Esq., Corresponding Member of the Medico-Botanical Society.

(Concluded.)

*Heart's-ease.* There is an interesting, and as I am informed an ancient custom, which has descended to the present day, now existing in some parts of Wales, that, when a lady wishes to deviate from the usual practice of waiting for certain advances to be made by the other sex, she, in a graceful and elegant manner, by presenting the gentleman with a flower of the Viola tricolor, is understood to make the first overture; and thus silently, but expressively, relief is made to supersede the anxiety of mind which is occasioned by a state of uncertainty and inquietude. Hence the name of Heart's-ease.

> "Musicians, O musicians! *Heart's-ease, Heart's-ease!* Oh,
> An you will have me live, play *Heart's-ease.*"—
> "Why Heart's-ease?"—
> "Oh, musicians, because my heart itself plays
> '*My heart is full of woe!*'"—*Romeo and Juliet,* act iv. sc. 5.

The French word *pensez,* supposed to be pronounced by the flower at the moment it is presented, as if conscious of the tale it bears, is the origin of our Pansey. Nothing can be more poetical than Shakspeare's use of this all but innocent flower, in his *Midsummer Night's Dream,* from which we naturally derive another etymology, that of

> "These blue-veined *violets* whereon we lean."—*Venus and Adonis.*

> "I saw, but thou couldst not,
> Cupid all armed: a certain aim he took
> At a fair vestal, throned by the west,
> And loosed his loveshaft smartly from his bow
> As it should pierce a hundred thousand hearts;
> But I might see young Cupid's fiery shaft
> Quench'd in the chaste beams of the watery moon,
> And the imperial maiden passed on,
> In maiden meditation, fancy free.
> Yet mark'd I where the bolt of Cupid fell;
> It fell upon a little western flower,
> Before milk white, now purple with Love's wound,
> And maidens call it *love in idleness.*
> Fetch me that flower, the herb I shew'd thee once;
> The juice of it on sleeping eyelids laid,
> Will make or man or woman madly dote
> Upon the next live creature that it sees. * * *
> And ere I take this charm off from her sight,
> (As I can take it with *another herb,*)
> I'll make her render up her page to me."—Act ii. sc. 2.

The medical efficacy of these plants, however, as cordials, would doubtless be lost in the form of syrup, notwithstanding its sweetness; and I have chiefly alluded to them for the purpose of inquiring what was the plant intended by Shakspeare, under the name of *Dian's bud,* to counteract the charming influence of *Love in idleness.*

"Dian's bud o'er Cupid's flower
Hath such force and blessed power."—Act iv. sc. 1.

Could this by possibility have been the Samolus valerandi? My curiosity once prompted me, when walking with a farmer of this neighbourhood, to ask him if he had a name for this venerated plant, and he informed me he had only once heard it called *Kenningwort* by an Englishman, in Wales, who performed remarkable cures as an oculist by its means. He said the plant received its name from its use in curing that complaint of the eye denominated "the Kenning," which is "when a substance resembling a pea forms upon the candle of that organ." Hence the Samolus, stated by Pliny to have been worshipped in this island by our ancestors, was probably this plant, and considered by them as emblematical of the efficacy of science in deterging from the intellect the foul cataracts of ignorance and error. How appropriate to the purpose of the poet! to dissipate by its agency the hallucination of love, and to dispel all overweening fondness for our most darling prejudices.

*Yew.* The yew seems to have taken its name from its having been employed in the construction of yokes for cattle; or perhaps, *vice versa,* the yoke, from its having been made of yew. In this latter case, the name would be derivable from the fruit, resembling in its form and in its viscous quality the yolk of an egg. Perhaps the name exists in the Greek *ζυγια,* our Carpinus, or true Welsh hasel, the workers of which were the original carpenters. On the Mediterranean shores, the cypress is used for coffins, because of its incorruptibility, and the tree is planted over the graves of the dead.

"Come away, come away, Death,
And in sad cypress let me be laid."—*Twelfth Night,* act ii. sc. iv.

"Cyprus black as e'er was crow."—*Winter's Tale,* act iv. sc. 3.

Again,

"Their sweetest shade a grove of cypress trees."
"A cyprus, not a bosom, hides my poor heart."—*Tw. N.* ac. iii. s. 1.

The true English name for the Tamaox gallica is Cifris, evidently similar to cypress, derived from a Hebrew word for grave, which occurs in the name of a station in Arabia, mentioned in the Pentateuch, *Kibroth hataavah,* or the "glutton's graves." The word *crape* is the English radix, confounded in orthography with the tree in the above passages. In the north of Europe the yew is planted for the same reason; its boughs wave over the hearse, and its sprigs are introduced into the coffin. Shakspeare, in his *Twelfth Night,* directs the shroud to be stuck with it.

> "My shroud of white, stuck all with yew,
> Oh, prepare it."—Act i. sc. 4.

The chemical principle upon which depends the incorruptibility of this beautiful wood, and which renders the tree all but immortal, is probably the same with its poisonous quality, and which rendered it an important ingredient in the witch's cauldron:

> "Gall of goat, and slips of yew,
> Stiver'd in the moon's eclipse."—*Macbeth,* act iv. sc. 1.

I understand, in some parts of England, it is the custom as soon as a person dies, to sponge the corpse over with infusion of its fresh leaves: this preserves the body from putrefaction, and preserves it for many weeks. Professor Martyn describes the case of a young lady, who was accidentally poisoned from drinking this infusion by mistake, instead of *rue* tea, as she was advised. The result was, that although dead, she retained the bloom of her countenance, so that her attendants believed her to be only in a trance: she was accordingly kept a long while uninterred, and was finally buried without any appearance of putrefaction. The importance of this wood in ancient warfare has suggested the epithet of double-fatal, used by our author in *King Richard II.,* act iii. sc. 2.

> "The very beadsmen learn to bend their bows
> Of double-fatal yew against thy state."

Some have supposed that it was on this account so highly venerated by our ancestors, and planted by them in our churchyards; but I consider this opinion to be unfounded. Many of the yew-trees of this country are certainly 3000 years of age, and I believe that most of those in our churchyards, which are four feet thick, and some are from eight to twelve, must be older than the introduction of Christianity into this kingdom; but the demonstration and the store of facts which corroborate and prove my position, are too copious to detail in this place.

*Plantage.* "As true as steel as plantage to the moon." Can this be the Alisma? or is it one of the Lunarias or moonworts? I suppose it is the Alisma plantago. The dedication of this herb to the moon, or Diana, from its temperature being considered cold, and from its influence upon hydrophobic patients and lunatics, and also from its seeds being emmenagogue, leads me to conjecture that this must be the species chosen by our poet as the emblem of fidelity. The word

Plantago, Plantage, or Plantain, implying the similarity of the leaf in shape to the sole of the foot, may be more strictly applicable to the Plantago major, or the sweet-scented media; but the virtues of all are very imperfectly known to the scientific world. An individual who was bitten by a mad cat was not affected with a dread of water till the lapse of a long period of time, but she experienced a recurrence of pain and irritation *at every change of the moon,* and she was finally attacked by death, after the regular intermission of a month. I am therefore of opinion that Shakspeare was acquainted with the fact that hydrophobia is relieved by the Alisma. It might be advantageous to our excellent Society, if its learned members were to institute inquiries amongst the poor people of the country, relative to the properties of this and such like plants, and not reject hastily and with disdain the important knowledge to be sometimes derived from their experience. It was by such inquiries that my late immortal friend made the discovery of vaccination, which, by philosophical reasoning and induction, he rendered more and more certain as a preventive of one of the most distressing "ills that flesh is heir to." The more common use of ordinary plantain as an application to wounds is likewise noticed by Shakspeare.

"*Benvolio.* Take thou some new infection to thy eye,
And the rank poison of the old will die.
*Romeo.* Your plantain leaf is excellent for that.
*Benvolio.* For what, I pray you?
*Romeo.* For your broken shin."—*Romeo and Juliet,* act i. sc. 2.

I cannot refrain from mentioning here two instances of the use of medico-botanical knowledge. The first is the case of an acquaintance of mine who has long been a cunning workman in British fancy woods: he walks rather lame, but occasionally goes a considerable distance. He broke his leg, and was confined to his chamber for four years. He informs me that the faculty considered his case as desperate, after trying all their skill without effect. He was recommended to a certain plant, which he was directed to apply externally as a poultice. This succeeded; it brought away splinters of bones, &c., and effected so complete a cure, that in a few weeks he was enabled to walk and enjoy exercise in the open air as before. It is curious that he defied me as a professor of botany to mention the name of the plant which had afforded him relief; he kept it secret, and assured me he had operated in the same successful manner upon four other persons discharged as incurable from our infirmary. In his chuckling he said that nobody had ever guessed at the remedy but a Frenchman, who declared that the peasants of that country were well acquainted with its merits. He was perfectly astonished when I told him it could be no other than Comfrey, and he immediately waved his hat in triumph, shouting "Comfrey for ever!"

The second was the daughter of a most respectable bookseller, who was confined with a slight sprain in her ancle, from dancing. The medical gentlemen were of great eminence who attended her; the injury became worse and worse; and, after a confinement to her bed of, I believe, eleven months, her life was almost despaired of, when permission was obtained to apply, at the suggestion of a poor individual, the simple leaf of a garden-herb, which was considered as likely to be of service. The leaf was applied, and from that moment the pain diminished. In a few days the leg was healed up, and the young lady completely restored to her health. A small specimen of the plant was carefully cultivated in a flowerpot, and when I was applied to for its name, I recognised the Valeriana Phu. In this manner I think it advisable to make inquiries, if opportunities should occur, respecting the Lunarias, or moonworts, and the Alyssums of Dioscorides, Pliny, Galen, and Tabermemontanus; comparing them with the criticisms of botanists, and the experience of country people. I have no doubt that much information would be obtained confirmative of the interesting anecdote related by your lordship.

The Mercurialis perennis, or Cynocrambi, called Dog's mercury, as is reported, from its virtue having been discovered by Hermes, is the plant used in Spain for curing hydrophobia. It is here called Bristol-weed, and, from its appearing to contain a blue dyeing and cathartic principle, similar in all probability to that of oleander and indigo, I suspect it to be the Glastum, or woad, of our ancestors. Glas is the Welsh word for what is blue and transparent; it occurs seemingly in the word Ganderglasses, or Gandergaws; our name for orchis, the first part of the word *Gander*, being merely the Greek ανηρ, and the Latin *anser*, meaning male, and therefore applicable to the O. mascula and O. morio, the flowers of which in pastures appear like gems, constituting a *gawdy* and brilliant enamel, one of the most beautiful in nature.

The plant Horehound is likewise a remedy for hydrophobia, and takes its name accordingly; the first syllable of the word being the Greek ωρεω, *curo;* and Diascorides attests its efficacy. The true horehound is therefore the Marrubium Alyssum, or the Alyssum of Galen.

We may likewise inquire relative to the Dog-violet, whether the Viola canina may not possess this faculty, or the Dentaria, called toothed-violets, and sometimes dog's-tooth violets, or the Erythronium dens canis; called also in Hampshire, where it is abundantly wild, the dog's or hound's tooth violet.

The name of hound's-tree, or hound's or dog's berry, given to the Cornus, would authorize a trial of this fruit likewise in this horrible complaint.

Whether the word Madnep indicate another cure may be inquired, and whether the name apply to Heracleum Sphondylium, Heracleum Panaces, Partinaca Opopanax, or the Angelica Archangelica?

But, if we are to believe Pliny, we are indebted to the oracle for the discovery that the root of Cynosbatos, or Cynorrhodon, Rosa canina, or dog-rose, is a remedy for hydrophobia. It will appear, from the following extracts, that our medical roses were all known to Shakspeare, and celebrated by him, although not indeed for their therapeutic efficacy.

Notwithstanding the best conserve is made from the fruit of the Rosa arvensis, or apple-rose, yet we must suppose that our poet, by scarlet hips, referred to the fruit of the common briar. "The oaks bear mast, the briers scarlet hips." *Timon*, act iv. sc. 3.

The R. damascena is used for syrups, and the variety γ of Martyn must be the true damask-rose, so elegantly and poetically referred to in the following passages:

> "Fair ladies mask'd are roses in the bud;
> Dismask'd, their damask sweet commixture shews,
> Are angels veiling clouds, or roses blown."—*Love's L. L.* a. v. s. 2.

> "There was a pretty redness in his lip,
> A little riper and more lusty red
> Than that mixed in his cheek: 'twas just the difference
> Betwixt the constant red and the mingled damask."—
> *As You Like It*, act iii. sc. 5.

> "I have seen roses damasked red and white,
> But no such roses see I in her cheek."—*Sonnet* 130.

> "Gloves as sweet as damask-roses."—*Winter's Tale*, act iv. sc. 3.

He also contrasts the *red* with the *white* rose, both of which contain the same medical astringent property. The white rose being rubbed on alkaline paper, instantaneously produces a very beautiful yellow colour, which may be used as a dye.

